# Categorising cheetah behaviour using tri-axial accelerometer data loggers: a comparison of model resolution and data logger performance

**DOI:** 10.1186/s40462-022-00305-w

**Published:** 2022-02-05

**Authors:** Natasha E. McGowan, Nikki J. Marks, Aaron G. Maule, Anne Schmidt-Küntzel, Laurie L. Marker, David M. Scantlebury

**Affiliations:** 1 School of Biological Sciences, Queen’s University Belfast, 19 Chlorine Gardens, Belfast, BT9 5DL UK; 2 Cheetah Conservation Fund, PO Box 1755, Otjiwarongo, Namibia

**Keywords:** Cheetah, Accelerometry, Behaviour classification, Random forest, Accelerometer performance, H2O package

## Abstract

**Background:**

Extinction is one of the greatest threats to the living world, endangering organisms globally, advancing conservation to the forefront of species research. To maximise the efficacy of conservation efforts, understanding the ecological, physiological, and behavioural requirements of vulnerable species is vital. Technological advances, particularly in remote sensing, enable researchers to continuously monitor movement and behaviours of multiple individuals simultaneously with minimal human intervention. Cheetahs, *Acinonyx jubatus*, constitute a “vulnerable” species for which only coarse behaviours have been elucidated. The aims of this study were to use animal-attached accelerometers to (1) determine fine-scale behaviours in cheetahs, (2) compare the performances of different devices in behaviour categorisation, and (3) provide a behavioural categorisation framework.

**Methods:**

Two different accelerometer devices (CEFAS, frequency: 30 Hz, maximum capacity: ~ 2 g; GCDC, frequency: 50 Hz, maximum capacity: ~ 8 g) were mounted onto collars, fitted to five individual captive cheetahs. The cheetahs chased a lure around a track, during which time their behaviours were videoed. Accelerometer data were temporally aligned with corresponding video footage and labelled with one of 17 behaviours. Six separate random forest models were run (three per device type) to determine the categorisation accuracy for behaviours at a fine, medium, and coarse resolution.

**Results:**

Fine- and medium-scale models had an overall categorisation accuracy of 83–86% and 84–88% respectively. Non-locomotory behaviours were best categorised on both loggers with GCDC outperforming CEFAS devices overall. On a coarse scale, both devices performed well when categorising activity (86.9% (CEFAS) vs. 89.3% (GCDC) accuracy) and inactivity (95.5% (CEFAS) vs. 95.0% (GCDC) accuracy). This study defined cheetah behaviour beyond three categories and accurately determined stalking behaviours by remote sensing. We also show that device specification and configuration may affect categorisation accuracy, so we recommend deploying several different loggers simultaneously on the same individual.

**Conclusion:**

The results of this study will be useful in determining wild cheetah behaviour. The methods used here allowed broad-scale (active/inactive) as well as fine-scale (e.g. stalking) behaviours to be categorised remotely. These findings and methodological approaches will be useful in monitoring the behaviour of wild cheetahs and other species of conservation interest.

**Supplementary Information:**

The online version contains supplementary material available at 10.1186/s40462-022-00305-w.

## Background

Global biodiversity loss is one of the biggest crises currently threatening the natural world [1–3]. Approximately 40% of plant species, 23% of invertebrates, and 18% of vertebrates assessed are considered to be threatened [4]. For mammals, 22% of described species [4] and 26% of assessed carnivores are considered to be threatened [5]. Some of the primary threats to carnivores include reductions in prey [6] and habitat [7], human-wildlife conflict (primarily in terms of livestock losses) [8–11], and illegal trade in animals or animal parts [11–15]. Conservation is therefore at the forefront of policy-making decisions worldwide [16–19].

To implement effective conservation and species management strategies, an understanding of target species populations, ecology, and behaviour is important to provide an insight into the status of the species, its needs, and putative causes of decline. When monitored over time, population data can indicate trends in particular groups of animals or in species as a whole [20, 21] or the efficacy of conservation efforts in comparison to control areas [22]. Remote sensing technologies such as camera traps have aided in species population assessments [23–26], as well as in our understanding of species ecology, and behaviour [23]. Advances in Global Positioning System (GPS) devices have further contributed towards understanding species ecology by providing insights into movements and habitat use. Knowledge of behaviour in space and time can provide insights into the importance of particular habitats and microhabitats for a species. For example, Wege et al. [27] identified novel foraging sites used by fur seals, contributing towards conservation policy-making as these sites were heavily utilised during the winter and were not previously considered when making assessments for potential marine protected areas (MPAs), where summer use is considered to be more important.

Accelerometer data loggers have been used independent of [28, 29] and in combination with [30–32] other remote sensing technologies such as GPS devices, magnetometers, and gyroscopes. Tri-axial accelerometers measure acceleration in three orthogonal axes (heave, surge, and sway), providing information on omnidirectional dynamic movement of an animal, as well as its posture (via static acceleration) [33, 34]. When accelerometers are used alongside devices such as GPS loggers, detailed behaviour patterns in space and time can be elucidated (e.g. [35, 36]). Unlike other remote sensing technology such as camera traps, loggers are fitted to the animals of interest (either directly or via collars or harnesses), providing data on the individual for the entire deployment period, not simply when activated. This feature is particularly useful for assessing the behaviours of cryptic species with large home ranges or that utilise difficult-to-monitor habitats (e.g. dense forests/bush, burrows, or expansive deserts). Although, the relative affordability and ease with which loggers can be deployed has led to their widespread use, less consideration appears to be given to device selection and subsequent downstream data processing. Most applications of animal-borne accelerometers have been to examine behaviours (e.g. [31, 37–40]), with several resulting in the categorisation of coarse-scale descriptions (i.e. three or four different behaviours) [28, 33, 40, 41]. While several studies have categorised behaviours manually by coarsely examining the acceleration traces generated (e.g. [33, 42]), others have implemented machine-learning techniques (many described in [43]), including random forests (RFs) [29, 31, 37–40, 43, 44], to classify behaviours to datasets using training and test data. Other approaches, such as the use of magnetometers, have proven successful in the determination of specific behaviours (e.g. biting and chewing in grazing herbivores) [45].

Cheetahs (*Acinonyx jubatus*) are medium-large felids inhabiting Africa and Iran [46–48]. They are classified as ‘Vulnerable’ by the IUCN [46] with the most recent population assessment (2014) suggesting just under 7100 adolescent and adult cheetah remain in the wild [47]. Population strongholds exist in southern and eastern Africa [46, 47]. Whilst conservation measures such as confiscation of traded animals and parts and reducing conflict with humans have been put in place, cheetah populations continue to decrease, with habitat loss, persecution, and illegal hunting and trade comprising major threats [7, 11, 47]. As such, detailed monitoring of cheetah movements, habitat use, and behaviour can assist with conservation efforts to ensure stringent monitoring of frequently used areas to reduce poaching and the adequate provision of resources to meet the needs of the species. To date, only coarse behaviours (active, inactive, and feeding) have been defined for cheetahs using remote sensing technology (accelerometers) [33, 41]. However, other ecological information such as different hunting strategies they may adopt and the associated costs of chasing prey [30, 32, 49, 50] (using GPS and accelerometers) have also been elucidated. However, while fine-scale behaviours, such as stalks (which may not result in a hunt), different movement gaits (e.g. walking vs. sprinting), and resting, have yet to be described for cheetahs, such data are available in other species (e.g. [31, 37–39, 43, 44]). Cheetahs are considered to be “extreme” movers, potentially reaching top speeds of 64mph (103kph) in a matter of seconds [51]. Therefore, the ability to distinguish between fine-scale behaviours may help to define the ecological needs of cheetahs, including hunting success rate, and, thus, contribute to conservation efforts. However, due to the high power and accelerations attained by cheetahs, monitoring their behaviour remotely may be limited by the capacity of individual devices.

The overall aim of the current study was to ground-truth behaviours performed by cheetahs against data collected using tri-axial accelerometers. Specifically, we wanted to (1) determine the accuracy with which a suite of behaviours in a cheetah’s repertoire could be defined; (2) determine whether this could be affected by the technical specifications of two different accelerometer devices, and; (3) provide a framework in the form of a vignette containing “R” code to develop behaviour categorisation models for other species of policy or conservation interest.

## Methods

### Study animals and collar preparation

This study was carried out in October 2012 at the Cheetah Conservation Fund (CCF) research centre near Otjiwarongo, Namibia (− 20.447763° N, 16.677918° E). Five resident adult cheetahs (three males and two females) were fitted with their own neck collars (nylon dog collars with plastic clip buckle: mass = 75 g, length = 570 mm, width = 20 mm) equipped with two tri-axial accelerometer data loggers: 1. G6, CEFAS Technology Limited, Lowestoft, UK (maximum = 2.3 *g*, size = 40 × 28 × 15 mm (L × B × D), mass = 18 g including urethane encasement, recording frequency = 30 Hz); 2. X8M-3, Gulf Coast Data Concepts (GCDC), LLC, Waveland, MS, USA (maximum = 8.6 *g*, resolution = 0.001 *g*, size = 50 × 30 × 12 mm (L × B × D), mass = 21.6 g including epoxy encasement, recording frequency = 50 Hz). To ensure the collar remained centred on the ventral side of the neck, an additional weight comprising four steel nuts (120 g) was added. The total weight of the fully equipped collars was approximately 235 g (see Additional file 3: Figure S1a for constructed collar design). Prior to being fitted to cheetahs, collars were hung on a metal rail with the accelerometers located at the bottom of the collar to allow for the devices to be calibrated (see “Data processing—accelerometers” below).

### Exercise arena and video capture

Cheetahs were exercised by chasing a lure (cloth rag) attached to ~ 285 m of cord around a pre-determined track. The lure machine, powered by an electric motor, was remotely controlled by a keeper, such that the speed and direction of the lure could be altered at will. The keeper changed the direction of the lure strategically to attempt to outwit the chasing animals and prevent capture of the lure. The chasing animals were thereby encouraged to employ different strategies to try to catch the lure, including stalking behaviour and high-speed pursuits. Each cheetah was exercised individually and behaviour was recorded using a video camera (Canon PowerShot SX230 HS; Canon, Japan). Typically exercise bouts lasted 10–15 min and consisted of three or four active chases (e.g. running, stalking) punctuated by two or three lower intensity rest periods (e.g. lying down, walking, standing). Collars were retrieved when the animal had finished exercising. As exercise bouts comprised periods of activity and inactivity, data associated with both hunting and resting were collected and ground-truthed against video footage.

### Data processing—accelerometers

Following exercise bouts, data loggers were removed from collars and data were downloaded. The data collected for both devices were calibrated to correct for non-centred mounting of the devices on the collars using the region of the dataset where the collars had been attached to the metal rail (see Additional file 1: Study details, collar calibration, and calculations). The data corresponding to the times of captured video footage were selected and the rest of the data were removed. Static acceleration (acceleration due to gravity; Additional file 5: Figure S2, static acceleration diagram) was derived for each axis from the corrected heave (acceleration in vertical axis), surge (acceleration in longitudinal axis), and sway (acceleration in transverse axis) data by calculating a rolling mean over a two-second window [52]. Dynamic acceleration was then calculated for each axis as the absolute result of subtracting static acceleration for a particular axis from its raw acceleration. Vectorial Dynamic Body Acceleration (VeDBA), Vectorial Static Body Acceleration (VeSBA), animal static acceleration (Anim.stat), pitch, and roll were also determined (Additional file 1: Study details, collar calibration, and calculations).

### Data processing—video footage

All video footage (approximately 58 min; 103,869 CEFAS logging events; 174,185 GCDC logging events) was synchronised with its complementary accelerometer datasets. Video footage was assessed frame-by-frame (Avidemux software; Developer: Mean) and cheetah behaviour was matched with the accelerometer data. Initially, 22 behaviours and behaviour combinations were identified (Table 1). Any other behaviour was recorded as ‘other’ and instances where behaviour could not be assigned (e.g. if an object obstructed a clear view of the animal) were removed from the dataset as we could not be certain of categorisation, resulting in a loss of approximately six minutes’ worth of data. Each labelled dataset was amalgamated to give two master spreadsheets of labelled accelerometer data; one for each model of accelerometer device (CEFAS and GCDC).

**Table 1:** Behaviours performed by cheetahs that were used to label accelerometer data

Behaviour	Description	Behaviour	Description
Crouch	Sedentary; ventral surface lowered to ground; feet parallel to body; forelimbs flexed	Stalk—crouching	Sedentary; crouched posture with head lowered, aligned with spine; attention focussed on lure
Lie	Crouched posture with forelimbs straightened OR sedentary; lateral surface on ground; head upright	Stalk—lying	Sedentary; lying posture with head lowered, aligned with spine; attention focussed on lure
Sit	Sedentary; posterior on ground; forelimbs straightened and anterior upright	Stalk—sitting	Sedentary; sitting posture with head lowered, parallel with or pointed towards ground; attention focussed on lure
Stand	Sedentary; only feet in contact with ground; limbs straightened and animal upright	Stalk—standing	Sedentary; standing posture with head lowered, aligned with spine or pointed towards ground; attention focussed on lure
Walk	Mobile; slow-paced movement; four-beat movement; all limbs move sequentially	Stalk—walking	Mobile; walking gait with head lowered, aligned with spine or pointed towards ground; attention focussed on lure
Flying Trot	Mobile; slow-paced movement; two-beat movement; diagonals appear to move in tandem; suspension period between diagonal movements; forelimb raised before ipsilateral hind limb lands	Stalk—trotting	Mobile; trotting gait with head lowered, aligned with spine or pointed towards ground; attention focussed on lure
Canter	Mobile; fast-paced; head and spine not aligned (head raised); asymmetric gait; three-beat movement (1-2-1); order = hind limb, diagonal limbs, forelimb	Eating—crouching	Sedentary; crouched posture whilst eating
Rotatory Gallop	Mobile; fast-paced; head and spine aligned; four-beat movement; trunk flexion and extension occurs; two suspensions, one after the second hind limb is raised and one after the lead forelimb is raised; order = sequential hind limbs, extended suspension, sequential forelimbs, collected suspension	Eating—lying	Sedentary; lying posture whilst eating
Bite	Head movement; Single movement of opening and closing mouth in an attempt to obtain food	Sniffing—sitting	Sedentary; sitting posture whilst sniffing
Eat	Head movement; Processing food already in mouth	Sniffing—standing	Sedentary; standing posture whilst sniffing
Sniff	Head movement; Head moves forward and upward whilst inhaling air through nose	Pounce	Single movement of an animal bounding off the ground in an attempt to catch the lure

Movement gaits are displayed visually in Fig. 4

### Data analysis

Data analysis was carried out in ‘R’ version 3.4.3 [54] using the ‘h2o’ package version 3.16.0.2 [53]. RF analysis (Additional file 2: Code) was conducted on the datasets labelled with behaviours. The datasets were split into three, such that 60% of cases were selected at random to entrain models (training dataset), 20% of cases were selected at random to validate the model (validation dataset), and the remaining 20% were used to test model performance (test dataset). The training data were used to entrain the RF model to categorise specific behaviours (see Table 2 for behaviour list). The validation dataset was then used to assess the performance of the model via model accuracy, (root) mean square error (RMSE and MSE), and r^2^. The validation data were also used to refine the model by altering model parameters and comparing the metrics listed above. The test data were only used once at the end of the process to compare model accuracy after validation to the outputs of the training dataset.

**Table 2:** Structure of fine-, medium-, and coarse-scale random forest (RF) behaviour models and frequency of occurrence of each

Behaviour	Behaviour model			Number of events	
	Fine-scale	Medium-scale	Coarse-scale	CEFAS	GCDC
Crouch	✓	✕	✕	638	1087
Lie	✓	✕	✕	32, 056	54, 544
Sit	✓	✕	✕	2389	4061
Stand	✓	✕	✕	13, 842	23, 536
Head movement	✓	✓	✓	1429	2405
Crouching stalk	✓	✓	✕	96	163
Lying stalk	✓	✓	✕	11, 692	19, 933
Sitting stalk	✓	✓	✕	119	204
Standing stalk	✓	✓	✕	1330	2201
Walking stalk	✓	✕	✕	3024	5132
Trotting stalk	✓	✕	✕	1267	2129
Walk	✓	✓	✕	6, 651	11, 161
Trot	✓	✓	✕	1295	2165
Canter	✓	✓	✕	3039	4985
Gallop	✓	✓	✕	4154	6976
Pounce	✓	✕	✕	87	141
Other	✓	✓	✓	20, 466	32, 549
Sedentary	✕	✓	✕	48, 925	83, 228
Moving stalk	✕	✓	✕	4291	7261
Active	✕	✕	✓	19, 517	32, 689
Inactive	✕	✕	✓	62, 162	105, 729

“✓” indicates inclusion of a behaviour to be categorised in a given model and “✕” indicates exclusion. Behaviour descriptions are provided in Table 1. “Head movement” = behaviours incorporating *biting*, *eating*, or *sniffing*; “Sedentary” = behaviours incorporating *crouching*, *lying*, *sitting*, or *standing*; “Moving stalk” = *Walking stalk* and *Trotting stalk*; “Active” = locomotory behaviours; “Inactive” = non-locomotory behaviours except *Head movement*

### Model structure

Initially, models were entrained to categorise 17 behaviours (Table 2, fine-scale). The predictor variables were: *heave*, *surge*, *sway*, *static heave*, *static surge*, *static sway*, *dynamic heave*, *dynamic surge*, *dynamic sway*, *VeDBA*, *VeSBA*, *Anim.stat*, *pitch*, and *roll*. A stopping criterion (stopping-rounds = 2) was implemented to optimise the duration for which models were run. A stopping criterion of two stops fitting the model when the two-tree average is within 0.1% accuracy of the previous two-tree average. If this criterion is increased, the average is taken over the specified number of trees. Models were refined by changing their depth and comparing their overall accuracy (percentage of correctly categorised behaviours divided by percentage of incorrectly categorised behaviours). The model with the highest accuracy was retained. Models were re-run using coarser behavioural categories (Table 2). For each model, cross-validation was performed using five folds and comparing mean accuracy, RMSE, MSE, and r^2^ to the training dataset. ‘R’ code for RF model constructs and additional model information are provided in the supplement (Additional file 2: Code). Logger performances were compared for the categorisation of each individual behaviour using chi-squared tests.

## Results

There was no indication of significant overfitting when cross-validation of models was carried out (Table 3).

**Table 3:** Mean cross-validation and training data metrics

Model	Device	Training				Cross-validation			
		Accuracy	RMSE	MSE	r^2^	Accuracy	RMSE	MSE	r^2^
Fine-scale	CEFAS	0.825	0.424	0.179	0.987	0.825 (0.005)	0.431 (0.004)	0.186 (0.003)	0.986 (< 0.001)
	GCDC	0.846	0.416	0.173	0.987	0.843 (0.003)	0.424 (0.001)	0.180 (0.001)	0.987 (< 0.001)
Medium-scale	CEFAS	0.838	0.398	0.158	0.982	0.837 (0.003)	0.404 (0.003)	0.163 (0.003)	0.981 (< 0.001)
	GCDC	0.852	0.394	0.155	0.982	0.849 (0.004)	0.401 (0.001)	0.161 (0.001)	0.981 (< 0.001)
Coarse-scale	CEFAS	0.877	0.327	0.107	0.885	0.876 (0.003)	0.329 (0.003)	0.109 (0.002)	0.883 (0.002)
	GCDC	0.894	0.312	0.097	0.894	0.890 (0.002)	0.319 (0.001)	0.102 (0.001)	0.889 (0.002)

Figures in brackets indicate standard deviation (n folds = 5)

### CEFAS loggers

**Fig. 1:**
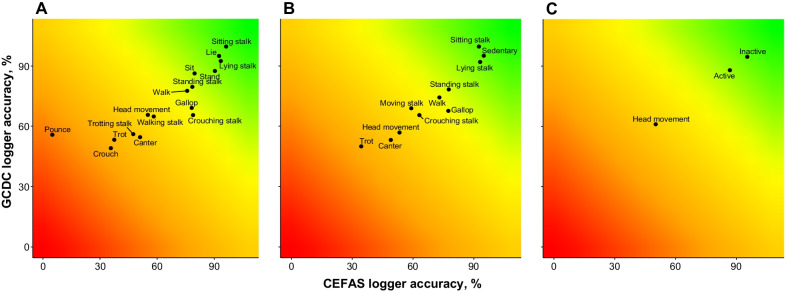
Heatplots of behaviour categorisation in fine-, medium-, and coarse-scale models. Behaviours in the green zone indicate those that were categorised well by both CEFAS and GCDC loggers, whereas behaviours in the red zone were categorised poorly by both

**Fig. 2:**
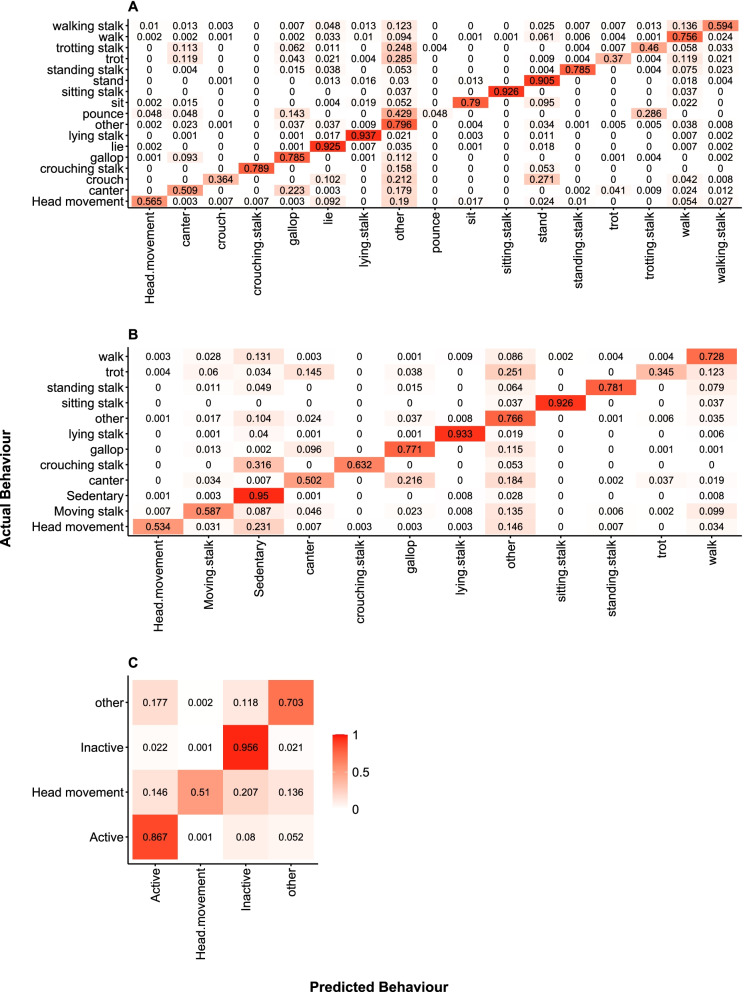
Confusion matrices for CEFAS accelerometer data loggers. **A** Confusion matrix for fine-scale behaviour model; **B** confusion matrix for medium scale behaviour model; **C** confusion matrix for coarse scale behaviour model. All matrices represent validation datasets. Darker shades of red represent higher classification values and shades that are more white indicate lower values. Values in each cell indicate frequency of classification

In the first model, behaviours were categorised on a fine scale. The behaviours sought to be categorised were: *crouch*, *lie*, *sit*, *stand, head movement*, *crouching stalk*, *lying stalk*, *sitting stalk*, *standing stalk*, *walking stalk*, *trotting stalk*, *walk*, *trot*, *canter*, *gallop*, *pounce*, and *other*. The overall accuracy of the model was 83.3% (MSE = 0.18, RMSE = 0.42, r^2^ = 0.99). However, as ‘*other*’ was an uninformative category, which didn’t require correct positive categorisation in the training dataset as it comprised a ‘rag bag’ of various movements, its categorisation could be disregarded (but the variable still remained in the model). Once disregarded, the accuracy of the model increased to 84.2%. S*itting stalk*, l*ying stalk*, *lying*, and *standing* were categorised with over 90% accuracy. Behaviours with < 50% categorisation accuracy included *pouncing*, *crouching*, *trotting*, and *trotting stalk* (see Table 4 ; Fig. 1 for full description of classification accuracy). *Crouching* behaviour was most often confused with *lying* (14.5%), *other* (27.6%), and *standing* (47.4%). *Trotting* and *trotting stalk* were most often confused with *cantering* (*trotting*: 21.1%; *trotting stalk*: 19.4%), *galloping* (*trotting*: 6.1%; *trotting stalk*: 13.2%), *other* (*trotting*: 42.2%; *trotting stalk*: 45.8%), and *walking* (*trotting*: 19.7%; *trotting stalk*: 9.7%). In addition, *trotting stalk* was confused with *walking stalk* (6.3%) (Fig. 2A). In terms of predictor variables, static acceleration in all three axes was most important in categorising behaviours (heave: scaled importance (improvement of MSE relative to maximum improvement across all predictors) = 100%, explanatory power = 14.4%; sway: scaled importance = 80.0%, explanatory power = 11.5%; surge: scaled importance = 71.1%, explanatory power = 10.2%), followed by VeDBA (scaled importance = 53.3%, explanatory power = 7.7%), roll (scaled importance = 52.0%, explanatory power = 7.5%), and heave acceleration (scaled importance = 51.6%, explanatory power = 7.4%). In all, these six predictors explained 58.7% of the RF model variance.

**Table 4:** Behaviour categorisation accuracy (%) for each accelerometer type and for each model resolution

Behaviour	CEFAS logger			GCDC logger		
	Fine-scale	Medium-scale	Coarse-scale	Fine-scale	Medium-scale	Coarse-scale
Crouch	35.6			49.3		
Lie	92.6			95.3		
Sit	79.7			86.6		
Stand	90.4			87.8		
Head movement	55.1	53.4	50.3	65.9	57.1	61.3
Crouching stalk	78.9	63.2		65.8	65.8	
Lying stalk	93.5	93.2		92.8	92.3	
Sitting stalk	96.3	92.6		100.0	100.0	
Standing stalk	78.5	77.7		79.9	78.6	
Walking stalk	58.2			65.1		
Trotting stalk	47.4			56.3		
Walk	75.8	73.1		77.9	74.6	
Trot	37.4	34.5		53.5	50.2	
Canter	51.0	49.1		54.8	53.4	
Gallop	78.2	77.5		69.4	67.9	
Pounce	4.8			56.0		
Sedentary		95.0			95.4	
Moving stalk		59.2			69.2	
Active			86.9			88.3
Inactive			95.5			95.0

“CEFAS” and “GCDC” indicate accelerometer types and “fine-scale”, “medium-scale”, and “coarse-scale” indicate model resolutions

In the second, coarser model, several behaviours from the previous model were combined in an attempt to reduce the error rate. ‘*Pounce*’ was entered as ‘*other*’ as it could not be categorised reliably and was often confused with several other behaviours. Behaviours in this model included: *Sedentary* (‘crouch’, ‘lie’, ‘sit’, ‘stand’), *head movement*, *moving stalk* (‘trotting stalk’, ‘walking stalk’), *crouching stalk*, *sitting stalk*, *lying stalk*, *standing stalk*, *gallop*, *canter*, *trot*, and *walk*. The overall accuracy of this model was 84.7% (MSE = 0.16, RMSE = 0.40, r^2^ = 0.98), which increased to 86.6% when ‘*other*’ behaviours were removed. *Sedentary*, *sitting stalk* and *lying stalk* were the only behaviours where the prediction accuracy surpassed 90%. The prediction accuracy for two behaviours was lower than 50%; *cantering* and *trotting* (see Table 4 and Fig. 1 for full description of classification accuracy). *Cantering* was most often confused with *galloping* (43.8%), *other* (34.4%), and *trotting* (8.0%), while *trotting* was most often confused with *cantering* (20.1%), *other* (40.9%), and *walking* (20.8%) (Fig. 2B). Once again, static accelerations (heave: scaled importance = 100%, explanatory power = 13.0%; surge: scaled importance = 87.8%, explanatory power = 11.5%; sway: scaled importance = 76.2%, explanatory power = 9.9%), VeDBA (scaled importance = 73.7%, explanatory power = 9.6%), and VeSBA (scaled importance = 50.8%, explanatory power = 6.6%) featured among the important predictor variables. Overall, the top five predictors explained 50.7% of the RF model variance.

The final, coarsest model comprised a simplified RF where behaviours were either deemed to be *active* (‘walk’, ‘trot’, ‘canter’, ‘gallop’, ‘walking stalk’, ‘trotting stalk’, ‘pounce’), *inactive* (‘crouch’, ‘lie’, ‘sit’, ‘stand’, ‘crouching stalk’, ‘lying stalk’, ‘sitting stalk’, ‘standing stalk’), *head movement*, or *other*. The accuracy of this final model was 88.2% (MSE = 0.11, RMSE = 0.32, r^2^ = 0.89). When ‘*other*’ was removed, model accuracy increased to 92.7%. *Inactivity* was predicted to the highest degree of accuracy and *head movement* with the lowest. *Activity* was predicted with 86.9% accuracy (see Table 4; Fig. 1 for full description of classification accuracy). Whilst *head movement* was most often confused with all three behaviour categories; *inactivity* (47.3%), *activity* (28.8%), and *other* (24.0%), *activity* was most often confused with *inactivity* (58.9%) and ‘*other*’ behaviours (40.2%) (Fig. 2C). The most important predictors in this model were VeDBA (scaled importance = 100%, explanatory power = 15.1%), static acceleration in the heave axis (scaled importance = 84.1%, explanatory power = 12.7%), and dynamic acceleration in the sway (scaled importance = 63.0%, explanatory power = 9.5%), heave (scaled importance = 56.8%, explanatory power = 8.6%), and surge (scaled importance = 52.5%, explanatory power = 7.9%) axes as well as static acceleration in the surge axis (scaled importance = 50.4%, explanatory power = 7.6%). In total, the top six variables explained 61.6% of the model variance.

### GCDC loggers

**Fig. 3:**
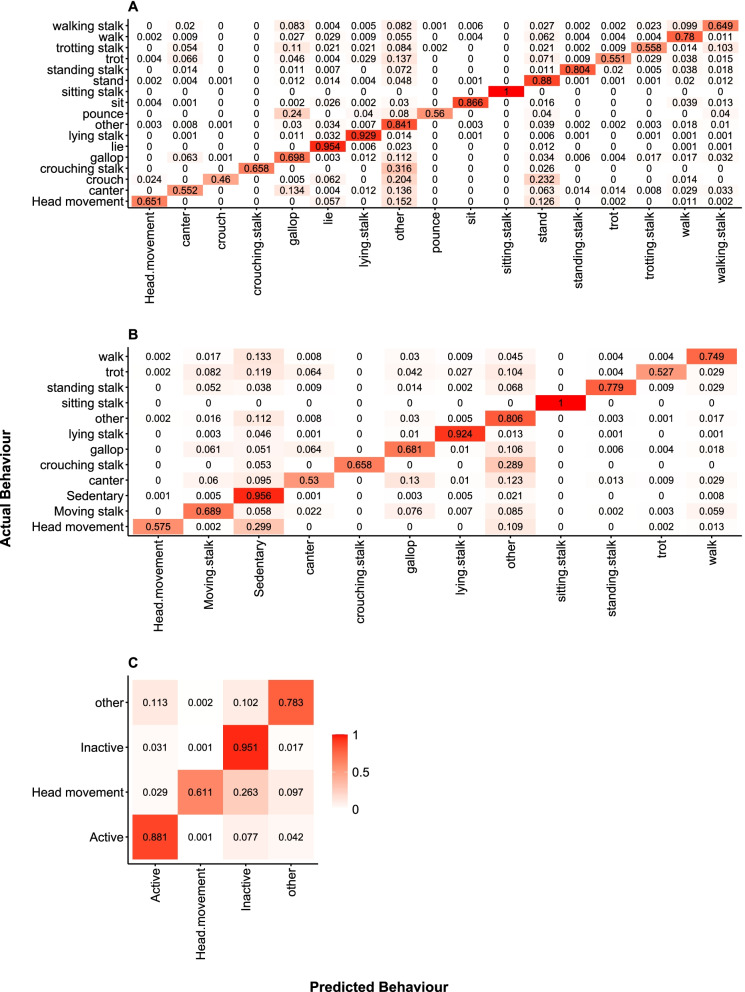
Confusion matrices for GCDC accelerometer data loggers. **A** Confusion matrix for fine-scale behaviour model; **B** confusion matrix for medium scale behaviour model; **C** confusion matrix for coarse scale behaviour model. All matrices represent validation datasets. Darker shades of red represent higher classification values and shades that are more white indicate lower values. Values in each cell indicate frequency of classification

The models outlined above were repeated for the GCDC data loggers. The model containing the finest-scale behaviours was 85.5% accurate (MSE = 0.17, RMSE = 0.41, r^2^ = 0.99). Accuracy increased to 85.8% when the category ‘*other*’ was omitted. The sedentary behaviours of *lying*, *lying stalk*, and *sitting stalk* were categorised with > 90% accuracy. *Crouching* was the only behaviour that was categorised with < 50% accuracy (see Table 4 and Fig. 1 for full description of classification accuracy); it was most often confused with *standing* (41.1%) and *other* behaviours (41.1%) (Fig. 3A). Static acceleration in all three axes was the most important predictor for behaviour (heave: scaled importance = 100%, explanatory power = 16.0%; sway: scaled importance = 80.1%, explanatory power = 12.8%; surge: scaled importance = 72.8%, explanatory power = 11.6%). In total, static acceleration variables explained 40.4% of the model variance.

When the second model outlined above was reproduced for the GCDC logger, it had an accuracy of 86.2% (MSE = 0.15, RMSE = 0.39, r^2^ = 0.98), which increased to 87.4% when the ‘*other*’ category was removed. Behaviours categorised with > 90% accuracy were *sedentary*, *lying stalk*, and *sitting stalk*. No behaviour categorisation was < 50% accurate; those behaviours that were most difficult to categorise were *trotting*, *cantering*, and *head movement* (see Table 4, Fig. 1 for full description of classification accuracy, and Fig. 3B for confusion matrix). In terms of predictor variables, static acceleration in all axes (heave: scaled importance = 100%, explanatory power = 14.7%; surge: scaled importance = 78.6%, explanatory power = 11.6%; sway: scaled importance = 78.2%, explanatory power = 11.5%), VeSBA (scaled importance = 51.8%, explanatory power = 7.6%), and Anim.stat (scaled importance = 51.5%, explanatory power = 7.6%) were most important in determining behaviours in this model. The top five predictor variables explained 53.1% of the model variance.

In the final RF model for the GCDC data loggers, *activity*, *inactivity*, *head movement*, and *other* behaviours were categorised. This model performed with a categorisation accuracy of 90.2% (MSE = 0.09, RMSE = 0.31, r^2^ = 0.90), which increased to 92.9% when the ‘*other*’ behaviour category was omitted. *Inactivity* was most easily classified and no behaviour had a classification accuracy below 61.3% (see Table 4, Fig. 1 for full description of classification accuracy, and Fig. 3C for confusion matrix). In this model, static acceleration in the heave (scaled importance = 100%, explanatory power = 12.7%) and surge (scaled importance = 84.8%, explanatory power = 10.8%) axes were most important for categorisation, followed by VeDBA (scaled importance = 83.2%, explanatory power = 10.6%), static acceleration in the sway axis (scaled importance = 75.8%, explanatory power = 9.6%), Anim.stat (scaled importance = 61.1%, explanatory power = 7.8%), VeSBA (scaled importance = 57.4%, explanatory power = 7.3%), and dynamic acceleration in the heave (scaled importance = 52.0%, explanatory power = 6.6%) and sway (scaled importance = 52.0%, explanatory power = 6.6%) axes. Together these eight variables explain 79.1% of the model variance.

### Logger comparison

Generally, the higher capacity loggers, with higher recoding frequency (GCDC) outperformed their lower capacity (CEFAS) counterparts when determining cheetah behaviour (Table 5; Fig. 1). This was particularly evident during medium- and fine-scale behaviour categorisation. Of the 30 different behaviour-model combinations run, the CEFAS loggers significantly outperformed the GCDC loggers only four times: *standing* (χ^2^ = 11.63, *df* = 1, *p* < 0.001) and *galloping* (χ^2^ = 19.46, *df* = 1, *p* < 0.001) in the fine-scale model, *galloping* (χ^2^ = 23.15, *df* = 1, *p* < 0.001) in the medium-scale model, and *inactivity* (χ^2^ = 4.42, *df* = 1, *p* = 0.035) in the coarse model (Table 5; Fig. 1). Conversely, the GCDC loggers were significantly better than the CEFAS loggers at behaviour categorisation on 11 occasions (Table 5; Fig. 1), notably when defining trotting, moving stalks, head movement, pouncing, and, in the fine-scale model, most sedentary behaviours. Whilst the GCDC loggers were better overall at defining behaviours on a fine- and medium-scale, there was no significant difference between the two devices when categorising behaviours on a coarse-scale (Table 5).

**Table 5:** Comparison of device performance (percent difference) for each behaviour in fine-, medium-, and coarse-scale models

Model	Behaviour	Percent difference (CEFAS-GCDC)	χ^2^	*df*	*p*	Better performing logger
Fine-scale behaviour model	Crouch	− 13.7	5.21	1	0.022	GCDC
	Lie	− 2.7	52.85	1	< 0.001	GCDC
	Sit	− 6.9	10.37	1	0.001	GCDC
	Stand	+ 2.6	11.63	1	< 0.001	CEFAS
	Head movement	− 10.8	8.51	1	0.004	GCDC
	Crouching stalk	+ 13.1	0.51	1	0.474	n.d
	Lying stalk	+ 0.7	1.14	1	0.286	n.d
	Sitting stalk^a^	− 3.7	0.00		0.380	n.d
	Standing stalk	− 1.4	0.13	1	0.722	n.d
	Walking stalk	− 6.9	7.52	1	0.006	GCDC
	Trotting stalk	− 8.9	4.92	1	0.027	GCDC
	Walk	− 2.1	2.00	1	0.158	n.d
	Trot	− 16.1	15.40	1	< 0.001	GCDC
	Canter	− 3.8	1.91	1	0.167	n.d
	Gallop	+ 8.8	19.46	1	< 0.001	CEFAS
	Pounce	− 51.2	11.40	1	< 0.001	GCDC
	Mean difference	− 6.4	20.62	1	< 0.001	GCDC
Medium-scale behaviour model	Head movement	− 3.7	0.84	1	0.360	n.d
	Crouching stalk	− 2.6	< 0.001	1	1.000	n.d
	Lying stalk	+ 0.9	1.57	1	0.211	n.d
	Sitting stalk^a^	− 7.4	0.00		0.141	n.d
	Standing stalk	− 0.9	0.03	1	0.872	n.d
	Walk	− 1.5	0.89	1	0.344	n.d
	Trot	− 15.7	14.88	1	< 0.001	GCDC
	Canter	− 4.3	2.52	1	0.112	n.d
	Gallop	+ 9.6	23.15	1	< 0.001	CEFAS
	Sedentary	− 0.4	2.25	1	0.134	n.d
	Moving stalk	− 10.0	23.95	1	< 0.001	GCDC
	Mean difference	− 3.3	5.52	1	0.019	GCDC
Coarse-scale behaviour model	Head movement	− 11.0	8.40	1	0.004	GCDC
	Active	− 1.4	3.11	1	0.078	n.d
	Inactive	+ 0.5	4.42	1	0.035	CEFAS
	Mean difference	− 4.0	0.87	1	0.350	n.d

Negative “percent difference” values indicate that GCDC devices performed best for categorising a given behaviour, whereas positive values indicate that the CEFAS logger was better

“n.d.”, no significant difference

^a^Fisher’s Exact test statistics (Odds ratio *in lieu* of χ^2^)

## Discussion

Recent, ongoing, and imminent species declines have prompted conservation focused research outputs (e.g. [55–58]). To make worthwhile steps towards successful conservation, it is important to not only know about populations and habitat requirements, but also about species’ behaviour, to ensure all ecological and biological needs can be met. To ensure that natural behaviours are not compromised, monitoring techniques should be minimally invasive [59], for example through the use of remote sensing technologies (such as lightweight GPS and accelerometer devices). Whilst the use of such devices has been gaining momentum for decades, interpretation of their outputs for behavioural categorisation is relatively recent, especially when high resolution and precision are desired (e.g. [31, 37–40]).

The cheetah (*Acinonyx jubatus*) is listed as ‘vulnerable’ [46] with wild populations purportedly decreasing [47]. However, descriptions of their movements and behaviour (particularly fine-scale behaviour) remain scarce [7, 30, 41, 49]. Accelerometry has been used to describe coarse behaviours in cheetahs; Grünewälder et al. [41] determined “mobile”, “stationary” and “feeding” with 84–94% accuracy and Shepard et al. [33] provide (without categorisation metrics) acceleration traces of walking, chasing, and trotting behaviours. In the current study, three RF models were constructed for fine-, medium-, and coarse-scale behaviour determination on high (“GCDC”, Maximum acceleration: ~ 8 g; Frequency: 50 Hz) and lower (“CEFAS”, Maximum acceleration: ~ 2 g; Frequency: 30 Hz) capacity devices. Using coarse modelling approaches (behaviours categorised as “Active”, “Inactive”, or “Head movement”.), RF analysis rendered consistent results (93% accuracy) between the two devices. Both devices categorised inactive behaviours well (GCDC = 95.0% accuracy and CEFAS = 95.5% accuracy), with the CEFAS logger performing best (Table 5). However, head movement could be described with just over 50% accuracy using CEFAS loggers (over 10% lower than GCDC devices), often confused with inactivity, which may be due to the core body remaining stationary. This finding suggests that even the use of collars does not guarantee reliable detection of head movement, which may, in fact, be beneficial if coarsely categorising behaviours. Head movement categorisation was better with GCDC devices (GCDC = 61.3% and CEFAS = 50.3% accuracy), which is probably due to their higher frequency recording so slight, short-lived movements were more likely to be detected [60]. In this model, dynamic acceleration (VeDBA and heave) was consistently important across both devices, which is unsurprising given the disparity in dynamic motion between the three categories. However, static acceleration in the heave and surge axes were also important parameters for the CEFAS logger, and several additional measures of static acceleration were important for the GCDC loggers (e.g. VeSBA), suggesting that postural changes may also play a significant role, especially as logger sensitivity increases. Practically, it is important to ensure consistent logger attachment, and device capacity and configuration should be considered when using results from previous studies to underpin novel research. The results of the current study are consistent with the only other study to categorise cheetah behaviour remotely using accelerometers [41]; “stationary” (“inactive”) behaviours were most accurately classified, followed by “mobile” (“active”) behaviours (Table 6). Feeding was specifically measured in this study rather than more generic “head movement” so the two categories may not be directly comparable. Classification of active behaviour was better in the current study and the overall performance was slightly better, which may be due to differences between the loggers used (bi-axial *versus* tri-axial), logger configuration, or analytical approach (SVM *versus* RF).

**Table 6:** Comparison of coarse behaviour model performance in the current study to Grünewälder et al. [41]

Modelled behaviour	Performance (percent correct)		
	Grünewälder et al. [41]	Current study—CEFAS	Current study—GCDC
Sedentary/inactive	97.2	95.5	95.0
Mobile/active	82.0	86.9	89.3
Feeding	71.4		
Overall	90.8	92.7*	92.9*

Provided are data for model performance (% categorised correctly) for each behaviour using GCDC and CEFAS accelerometers in the current study and mean performance of support vector machine (SVM) provided in Grünewälder et al. [41]. *Overall score also includes “Head movement”.

### Fine-scale behaviours

One objective of the current study was to determine whether fine-scale cheetah behaviours could be categorised using accelerometers. Such data could provide information on cheetah ecology and assist conservation efforts. For example, if foraging requirements (indicated by chases and stalks), the frequency of abandoned hunts (by identification of stalks with no subsequent pursuit), or changes in behaviour associated with life history such as rearing offspring could be identified accurately, specific ecological needs could be addressed by ensuring prey and habitat requirements were met. In the current study, a fine- and medium-scale behaviour categorisation model was produced for each accelerometer device; the finest-scale model included all behaviours that could be derived from video footage, whilst the medium-scale model collapsed several of these categories together, resulting in marginally coarser classification. Although both performed less well than coarse (active/inactive/head movement) models, there was little difference between the fine- and medium-scale models themselves. As such, it may be prudent to categorise behaviours on the finest or coarsest scales as they are more accurate (coarsest) or the benefit of additional behavioural information outweighs the marginal cost in accuracy (finest). To our knowledge, this study represents the most ambitious attempt to elucidate cheetah behaviour, with the highest resolution, fine-scale models incorporating 16 behaviours, and the coarser, medium-scale models including 11. Across both sets of models and both devices lying, lying stalks, and sitting stalks were always classified with over 90% accuracy; in fact, sitting stalks were classified with 100% accuracy on the GCDC loggers. This is the first time that these behaviours have been classified remotely with such accuracy in cheetahs. Stalks usually occur prior to pursuits of prey in cheetahs [61] so knowledge of the habitats that may facilitate stalks and successful hunts could be of great importance for survival. As such, acceleration data combined with GPS locations could provide vital insights for conservation. Furthermore, sedentary behaviours were categorised with a high degree of accuracy, which, when combined with other approaches, may provide insights into cheetahs’ physiological and habitat requirements.

**Fig. 4:**
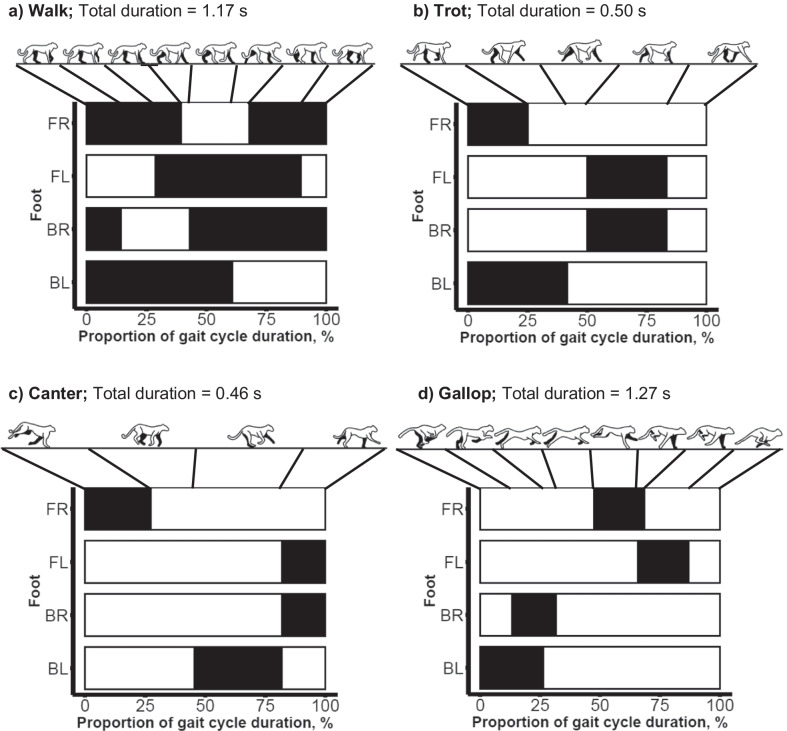
Depiction of locomotory gaits and relative duration of each footfall in the gait cycle. **a** Walking gait, **b** Trotting gait, **c** cantering gait, and **d** galloping gait. In each plot FR = front/fore right foot; FL = front/fore left foot; BR = back/hind right foot; BL = back/hind left foot. Shaded areas indicate when foot is in contact with the ground; unshaded areas indicate when foot is raised

The lowest (walking) and highest intensity (galloping) locomotory behaviours were categorised best with a higher error rate for intermediate trotting and cantering. Nevertheless, classification accuracy of walking and galloping was always between 68 and 78%. As footfall and rhythmicity of each locomotory gait varies (Fig. 4), incorporating periodicity may beneficial to differentiate them [28] but may be limited by rapid transitions between them and a lack of continuous measurements of any one in isolation. Correct identification of each gait could assist conservation efforts by providing insights into hunting and evasion, potentially facilitating the identification of areas favoured for hunting or resting, or those where cheetahs may be threatened by other species. Incorporation of lab-based techniques, such as indirect calorimetry, would allow us to determine the relative energetic cost of each behaviour and the overall proportion of their daily energy expenditure attributable to each [62]. Such an approach would inform management strategies, potentially reducing conflict with livestock owners [10].

Pouncing represented the worst categorised behaviour, with only 4.8% accuracy on the CEFAS logger (Table 4). This poor performance is likely due to a combination of low recording frequency, the instantaneous nature of the behaviour, and its rarity. However, pouncing is likely to be uncommon in free-ranging adult cheetahs, which primarily implement stalk-and-chase hunting strategies [61]. As such, the low classification accuracy in this context is not concerning but may be problematic when trying to define the behaviour in ambush hunters.

It is worth noting as a caveat that certain behaviours were underrepresented in the datasets e.g. pouncing and stalking, with some others overrepresented (e.g. sedentary behaviours such as lying). This imbalance may have affected how the data were split into training, validation, and test data and, ultimately, the models. However, with the approach taken here ecologically important behaviours such as stalking could be incorporated into the models and was likely to be randomly selected for a split based on its representivity. The result was reasonably reliable models (according to accuracy, MSE, RMSE, and r^2^) with several under-, over- and well-represented behaviours being predicted accurately,

### Application and experimental design

Generally, there was good consistency in model accuracy between CEFAS and GCDC accelerometer data. However, it is important to note that whilst CEFAS loggers categorised more behaviours with > 90% accuracy (*n* = 8; Table 4), than the GCDC loggers (*n* = 7), the latter categorised fewer behaviours with < 50% accuracy (GCDC: *n* = 1; CEFAS: n = 6). It is therefore important to determine *a priori*, where possible, the scale at which behavioural categorisation is desired and select devices and analytical models accordingly.

Whilst reliable categorisation was established for some cheetah behaviours, the GCDC logger outperformed the CEFAS logger in both fine- and medium-scale models (Table 5). Two potential reasons may explain this better performance: GCDC loggers could record higher accelerations (~ 8 g *versus* the ~ 2 g capacity of the CEFAS loggers) and were set to record at higher frequencies (50 Hz *vs.* 30 Hz). During high intensity galloping the CEFAS loggers reached maximum capacity, which may have led to a high frequency of correct categorisation for this behaviour but it may also have contributed to a high false positive rate for other, relatively high intensity behaviours such as trotting. Whilst locomotory behaviours were most often confused with adjacent behaviours for both loggers (e.g. cantering was most likely to be confused with trotting or galloping), trotting, cantering, and trotting stalks were the only locomotory behaviours identified with < 50% accuracy (occurring on the CEFAS loggers). Recording frequency may be important as higher logging rates will generate more data, rendering more information for entrainment of RF models. Recording frequency may be particularly important for rarely occurring and short-lived behaviours such as pouncing. Whilst a multitude of variables were used to entrain RF models, it is possible that others may also assist in categorising behaviours, for example, periodicity (movement rhythmicity) may be useful in discriminating between various locomotory gaits [28]. As there were significant performance differences between the two devices, there is an onus on researchers to select those with an appropriate specification for their study species, or, indeed, to use multiple different loggers in tandem on the same individual.

Behaviours have previously been categorised using accelerometer data loggers without the use of complementary video capture in cheetahs [33] and other species [42]. In such studies, behaviours are usually differentiated via variations in dynamic body accelerations and posture. However, if such an approach were implemented here, fine-scale sedentary behaviours would have been erroneously categorised, resulting in misinterpretation of species behavioural ecology. For example, resting behaviours such as standing or lying down could have been confused with sedentary stalks, where the former would signify true resting but the latter would indicate an attempted hunt. It is therefore recommended that data collected on accelerometer devices are synchronised with an extensive behavioural repertoire for the species.

## Conclusions

In this study we found that the ability to categorise behaviours differed significantly between data loggers. The results of the current study can be used to form the basis of remotely monitoring coarse- and fine-scale behaviours of the vulnerable cheetah. Knowledge of their behaviours can inform cheetah biology and ecology, particularly when combined with other loggers such as GPS. Once the basic needs of the cheetah have been firmly established, the efficacy of conservation and management practices can be maximised, and strategies can be implemented to mitigate human-cheetah conflict. The approach taken here may be adopted in remote-sensing studies of other species but careful consideration of logger capacity and recording frequency is recommended.

## Supplementary Information

**Additional file 1:** Supplementary information on study design, collar calibration and calculations.

**Additional file 2:** R code vignette to categorise behaviours using Random Forest analysis.

**Additional file 3:** Diagrammatic representation of collar design. **A** = collar with buckle in black; **B** = devices (see C for detailed descripton); (+) = positive acceleration in given axis; (-) = negative acceleration in given axis; Inset C: grey shaded areas = steel nuts; acc = accelerometer devices stacked atop each other; s.a.t. = self-amalgamating tape, securing nuts and devices to collar; t.t. = Tesa tape, adding additional security and protection to devices.

**Additional file 4:** Aerial view of the arena in which cheetahs were exercised. Black line = course on which lure travelled; brown circles = termite mounds; grey lines = perimeter fences.

**Additional file 5:** Graphic indicating the change in static acceleration (grey arrow). **a**) Cheetah standing upright with static acceleration affecting the heave (vertical) axis e.g. giving a value of 1 *g*. **b**) Cheetah with head pointed towards the ground, resulting in static acceleration registering in the surge (longitudinal) axis e.g. giving a value of 1 *g*. **c**) Cheetah lying on back with static acceleration affecting  the heave (vertical) axis e.g. giving a value of -1 *g *(opposite of scenario a).

## Data Availability

All data generated or analysed during this study and analysis code are included in supplementary information files.

## Abbreviations

CEFAS: Centre for Environment, Fisheries and Aquaculture Science
GCDC: Gulf coast data concepts
GPS: Global positioning system
RF: Random forest
CCF: Cheetah conservation fund
VeDBA: Vectorial dynamic body acceleration
VeSBA: Vectorial static body acceleration
Anim.stat: Animal static acceleration
MSE: Mean squared error
RMSE: Root mean square error
SVM: Support vector machine
