# Supplementary material for: Categorising cheetah behaviour using tri-axial accelerometer data loggers: a comparison of model resolution and data logger performance
Source: Mov Ecol. 2022 Feb 5;10:7. doi: 10.1186/s40462-022-00305-w (PMC8818224; doi:10.1186/s40462-022-00305-w)
Supplement: Supplementary file 1 — Additional file 1: Supplementary information on study design, collar calibration and calculations. [file 40462_2022_305_MOESM1_ESM.docx]

**Supplementary Information – R Code**

Random forest (RF) analysis is a useful, bootstrap aggregating (bagging), ensemble technique that facilitates the categorisation of information, such as behaviours, from a series of inputs [S1] and H_2_O is a software platform for handling large datasets and running such analyses. The following vignette describes a method by which behaviour categorisation can be achieved for accelerometer data loggers using random forest analysis in H_2_O (H2O.ai, CA, U.S.A.), run through R [S2]. The code for some of this analysis is available at [S3]. In order to execute the analysis, the following software is required: R (available from [S4]); RStudio (optional; available from [S5]); a compatible version of Java (available from [S6]).

Vignette

Before beginning the random forest (RF) analysis, ensure the accelerometer data have been calibrated for each axis [S7] and any information to be used in the RF models as explanatory (predictor) variables have been derived as appropriate (e.g. Vectorial Dynamic Body Acceleration (VeDBA), Vectorial Static Body Acceleration (VeSBA), Periodicity, etc.) [S8-9]. Each variable should be held in its own column in a datasheet; data should be stored in long format as opposed to wide format. For the purposes of the following vignette, a comma delimited datasheet has been used but other formats are also acceptable (e.g. tab delimited text). In addition to the raw and derived accelerometer data to be used as predictor variables, the datasheet should also contain a column of the behaviours, corresponding to the times at which each occurred. This can be achieved by capturing video footage of an animal while wearing an accelerometer data logger [S8, S10]. Behaviour labels should complement those that are to be described, for example, if periods of activity or inactivity are sought and the animal is walking, this should be labelled as “activity” in the dataframe. Once all data have been corrected and derived, and behavioural labels have been assigned, categorisation can begin.

The first step in behaviour categorisation is to remove old versions of the “h2o” package [S11] in “R” and to install the latest version of the package and its dependencies.

#Check if the "h2o" package has already been installed previously. If so, remove the package.

> **if(**"package:h2o" %in% search**())** **{**

detach**(**"package:h2o", unload**=T)**

**}**

> **if(**"h2o" %in% rownames**(**installed.packages**())){**

remove.packages**(**"h2o"**)**

**}**

#Install "h2o" from CRAN mirror and load.

> install.packages**(**"h2o"**)**

> library**(**h2o**)**

Once loaded, all current clusters should be stopped and a new cluster initiated.

#Initiate a new cluster

> localH2O **=** h2o.init**(**nthreads**=-**1**)**

#Clear data from existing clusters

> h2o.removeAll**()**

When a connection has been established, any executed code may be visualised online via the connection IP (localhost) and port (54321) (enter “localhost:54321” in your browser’s search bar). Next, load and parse the appropriate datasheet, and randomly assign 60% of the data to a file to entrain models (training data), 20% to validate and refine models (validation data), and the remaining 20% to test the final model (test data).

#Load file from disk and parse

> df **<-** h2o.importFile**(**path **=** "file pathway", parse**=**T**)**

#Create list of three splits - 60%, 20%, and remainder (20%)

#Set seed so that the results can be replicated if necessary

> splits **<-** h2o.splitFrame**(**df, c**(**0.6,0.2**)**, seed **=** 123**)**

#Assign first split (60%) to training data (train), second split (20%) to validation data (valid), and remainder (20%) to test data (test)

> train **<-** h2o.assign**(**splits**[[**1**]]**, "train.hex"**)**

> valid **<-** h2o.assign**(**splits**[[**2**]]**, "valid.hex"**)**

> test **<-** h2o.assign**(**splits**[[**3**]]**, "test.hex"**)**

The first predictive model can then be created and viewed (https://www.youtube.com/watch?v=9wn1f-30_ZY&t=2199s). Each of the model’s arguments is explained below the following code.

> rf **<-** h2o.randomForest**(** #Line 1

training_frame **=** train, #Line 2

validation_frame **=** valid, #Line 3

x **=** c**(**1**:**18**)**, #Line 4

y **=** 19, #Line 5

model_id **=** "RF1", #Line 6 (optional)

ntrees **=** 500, #Line 7

stopping_rounds **=** 2, #Line 8

score_each_iteration **=** T, #Line 9

seed **=** 25**)** #Line 10

#View model

> summary**(**rf**)**

Line 1: Create RF model called “rf”; Line 2: Input H_2_O frame with which to train the model; Line 3: Input H_2_O frame to validate and refine the model; Line 4: Vector (or names) of columns containing any predictor variables e.g. Accelerations (raw, dynamic, static) in each axis, VeDBA, VeSBA, etc.; Line 5: Response variable (Behaviour); Line 6: Name of the model in H_2_O; Line 7: Maximum number of trees to compute (default = 50); Line 8: Stops fitting the model when the two-tree average is within 0.1% (default) accuracy of the previous two-tree average. The figure denotes the number of trees to average across. If this level of accuracy is not achieved, the model will continue to run until the “ntree” limit is reached; Line 9: Predict against training and validation data for each tree; Line 10: Set random seed so that the model can be reproduced.

Refinement and improvement of RFs can occur by changing the number of trees (less applicable when “stopping_rounds” has been set), increasing the number of stopping rounds, changing the predictor variables, or increasing the depth of the trees (“max_depth = x”; where x = number denoting depth (default = 20)). Models can be compared by comparing their overall accuracies.

#Accuracy of first model using validation data

> h2o.hit_ratio_table**(**rf, valid **=** T**)[**1,2**]**

#Accuracy of new model using validation data

> h2o.hit_ratio_table**(**rf_new, valid **=** T**)[**1,2**]**

Once the most accurate model has been determined, it should be tested against the “test” dataset. For the model to be applicable to other datasets, there should be no appreciable difference between the accuracy of your validation and test data.

#Create model predictions against test dataset (instead of validation)

> finalRF_predictions **<-** h2o.predict**(**object **=** rf_new, newdata **=** test**)**

#Accuracy of model using validation data

> h2o.hit_ratio_table**(**rf_new, valid **=** T**)[**1,2**]**

#accuracy of model using test data

> mean**(**finalRF_predictions**$**predict **==** test**$**Behaviour**)**

Models can be saved for future use as binary files (not compatible across different versions of H2O) or MOJOs (compatible across different versions of H2O).

#Save model as binary overwriting previous files with same name (force = F to disallow overwrite)

> h2o.saveModel**(**rf_new, path **=** "pathway", force **=** **T)**

#Save model as MOJO overwriting previous files with the same name(force = F to disallow overwrite)

> h2o.save_mojo**(**rf_new, path **=** "pathway", force **=** **T)**

If an RF model has been saved, it can be reloaded at any time and applied to a new dataset where behaviours have not been validated against video footage. Datasets should contain the same column names as the dataset upon which the model was trained and tested, including a column labelled “Behaviour”. The “Behaviour” column in this dataset should be unpopulated. Behaviours can thus be predicted using the saved model and the accuracy of each prediction can also be determined.

#Load dataset in which behaviours are unknown and parse

> unk **<-** h2o.importFile**(**path **=** "pathway", parse**=**T**)**

#Load previously saved binary model

> rf**<-**h2o.loadModel**(**path **=** "pathway"**)**

#Load previously saved MOJO model

> rf**<-**h2o.import_mojo**(**path **=** "pathway.zip"**)**

#Predict behaviours for data using saved and loaded RF model

> preds **<-** h2o.predict**(**object **=** rf, newdata **=** unk**)**

#Output predictions as dataframe

> preds**<-**as.data.frame**(**preds**)**

#Load dataset in which behaviours are unknown but do not parse

> unknown **<-** read.delim**(**"pathway.csv", header**=**T, sep**=**","**)**

#Place predicted behaviours from model into "Behaviour" column of datasheet and change name to "Predicted behaviour"

> unknown**[**19**]<-**preds**[**, 1**]**

> colnames**(**unknown**)[**19**]<-**"Predicted Behaviour"

#Create an empty list

> result **=** vector**(**"list"**)**

#Loop through all of the rows of dataframe of predictions and populate the empty list with prediction accuracies (frequency)

> **for(**i **in** 1**:**nrow**(**preds**)){**

**if(**preds**$**predict**[**i**]** **==** "Behaviour1"**){**

result**[[**i**]]** **=** preds**$**Behaviour1**[**i**]**

**}** **else** **{**

**if(**preds**$**predict**[**i**]** **==** "Behaviour2"**){**

result**[[**i**]]** **=** preds**$**Behaviour2**[**i**]**

**}** **else** **{**

**if(**preds**$**predict**[**i**]** **==** "Behaviour3"**){**

result**[[**i**]]** **=** preds**$**Behaviour3**[**i**]**

**}** **else** **{**

**if(**preds**$**predict**[**i**]** **==** "Behaviour4"**){**

result**[[**i**]]** **=** preds**$**Behaviour4**[**i**]**

**}** **else** **{**

**if(**preds**$**predict**[**i**]** **==** "Behaviour5"**){**

result**[[**i**]]** **=** preds$Behaviour5**[**i**]**

**}** **else** **{**

result**[[**i**]]** **=** preds$Behaviour6**[**i**]**

**}**

**}**

**}**

**}**

**}**

**}**

#Bind the rows of the list of accuracies together

> result **=** do.call**(**rbind, result**)**

#Give new object a name

> Acc**<-**result**[**,1**]**

#Convert accuracies from a frequency to a percentage

> Acc**<-** Acc*****100

#Add accuracies to a new column of the datasheet containing predicted behaviours and rename column "Probability"

> unknown**[**20**]** **=** Acc

> colnames**(**unknown**)[**20**]** **=** "Probability"

#Write populated spreadsheet to designated pathway

> write.csv**(**unknown, "pathway.csv"**)**

**References**

[S1] Liaw A & Wiener M. Classification and Regression by randomForest. R News. 2002; 2/3: 18 – 22.

[S2] R Core Team. R: A language and environment for statistical computing, R Foundation for Statistical Computing, Vienna, Austria. URL: <https://www.R-project.org/>; 2017.

[S3] H2O.ai. <https://www.youtube.com/watch?v=9wn1f-30_ZY&t=2199s>. Accessed 25/09/2020.

[S4] <https://cran.r-project.org/mirrors.html>. Accessed 25/09/2020.

[S5] https://rstudio.com/products/rstudio/download/. Accessed 25/09/2020.

[S6] Oracle. <https://java.com/en/download/>. Accessed 25/09/2020.

[S7] GCDC (2020) Calibration instructions <http://www.gcdataconcepts.com/Calibration_Instructions.pdf>. Accessed 25/09/2020.

[S8] McClune DW, Marks NJ, Wilson RP, Houghton JDR, Montgomery IW, McGowan NE, et al. Tri-axial accelerometers quantify behaviour in the Eurasian badger (*Meles meles*): towards an automated interpretation of field data. Anim Biotelemetry. 2014; 2.

[S9] Bidder O, Walker JS, Jones MW, Holton MD, Urge P, Scantlebury DM, et al. Step by step: reconstruction of terrestrial animal movement paths by dead-reckoning. Mov Ecol. 2015; 3.

[S10] Halsey LG, Portugal SJ, Smith JA, Murn CP & Wilson RP. Recording raptor behavior on the wing via accelerometry. J Field Ornithol. 2009; 80: 171 – 7.

[S11] The H2O.ai Team. h2o: R interface for H2O, R package version 3.16.0.2. <https://CRAN.R-project.org/package=h2o>; 2017.
