# Supplementary material for: Categorising cheetah behaviour using tri-axial accelerometer data loggers: a comparison of model resolution and data logger performance
Source: Mov Ecol. 2022 Feb 5;10:7. doi: 10.1186/s40462-022-00305-w (PMC8818224; doi:10.1186/s40462-022-00305-w)
Supplement: Supplementary file 2 — Additional file 2: R code vignette to categorise behaviours using Random Forest analysis. [file 40462_2022_305_MOESM2_ESM.docx]

**Supplementary Information - Methods**

*Study animals and collar preparation*

In total, seven adult cheetahs (four males and three females) from two kin groups (A = two males, two females; B = two males, one female) were initially used in this study but one male from kin group A and the female from kin group B were omitted due to logger failure during deployment. The groups entered CCF as young cubs (A = approx. 1 month; B = approx. 2 days) and were 26 and 50 months old respectively. The individuals were therefore tractable and were habituated to wearing collars prior to this study. All cheetahs were fitted with two different collar-borne tri-axial accelerometers (CEFAS and GCDC loggers). First, the GCDC logger was affixed to the CEFAS logger using self-amalgamating tape (Ultratape; Bruce Douglas Marketing, Dundee, UK; 19mm×10m). The stacked loggers were then attached to the collar, adjacent to the buckle, using an additional layer of self-amalgamating tape, such that the GCDC device was on the bottom, in direct contact with the collar, and the CEFAS device was on top (not in direct contact with the collar). Once attached, the loggers were further secured to the collar using Tesa® tape (No. 4651; tesa AG, Hamburg, Germany). In order to ensure the collar remained centred on the ventral side of the neck, an additional weight comprising four steel nuts (120g) was added; two nuts were affixed on either side of the devices using self-amalgamating tape. The total weight of the fully equipped collars was approximately 235g (Fig. S1a for collar design).

*Exercise arena*

A 2.5 ha area consisting of level open scrub with several termite mounds and small trees was used to exercise the cheetahs (Fig. S1b). A cloth rag attached to ~285 m of cord was used as a lure. The lure machine, powered by an electric (car starter) motor, could travel along the ground in two directions around the course, and could reach maximum speeds of 70 km/h (19.44 m/s). The lure system was operated by a researcher using a remote control, which meant that the speed and direction of the lure could be altered at will. If a cheetah caught the lure during an exercise bout, it was rewarded with a small (~50 g) piece of fresh meat.

*Data processing - accelerometers*

For the GCDC devices, acceleration data were converted from count data to *g* by dividing by the appropriate scale factor (1024) [S1]. The data collected for both devices were calibrated to correct for non-centred mounting of the devices on the collars using the region of the dataset where the collars had been attached to the metal rail. Mean acceleration was calculated for this period for each axis (heave: acceleration in vertical axis; surge: acceleration in longitudinal axis; and sway: acceleration in transverse axis). For the surge and sway axes, the difference between zero and their respective mean accelerations was the correction factor for that particular axis, as they should not register any acceleration (static acceleration due to gravity or dynamic acceleration due to active movement) when at rest (Eq. 1). For the heave axis, the correction factor equalled the difference between one and the mean acceleration for the axis; when at rest, gravity should render an acceleration (static) of one in this axis but no dynamic acceleration should be present (Eq. 2). Corrections were applied to each axis by adding the appropriate correction factor to the entire dataset for the corresponding axis. The data corresponding to the times of captured video footage were then selected and the rest of the data were removed. Static acceleration (Fig. S2) was derived for each axis from the corrected heave, surge, and sway data. Dynamic acceleration was then calculated for each axis as the absolute result of subtracting static acceleration for a particular axis from its raw acceleration. Vectorial Dynamic Body Acceleration (VeDBA), Vectorial Static Body Acceleration (VeSBA), animal static acceleration, pitch, and roll were determined (Eq. 3-7 respectively).

$Corr=Acc+ \left( 0- \bar{x}{Acc}_{rest} \right)$ Eq.1

Where Corr = corrected sway or surge acceleration (*g*), Acc = Sway or surge acceleration value (*g*), x̅ = mean, Acc_rest_ = acceleration in sway or surge axis when the animal is at rest (*g*).

${Heave}_{corr}=Heave+ \left( 1- \bar{x}{Acc}_{rest} \right)$ Eq.2

Where Heave_corr_ = corrected heave acceleration (*g*), Heave = Heave acceleration value (*g*), x̅ = mean, Acc_rest_ = acceleration in heave axis when the animal is at rest (*g*).

$VeDBA= \sqrt{{Heave}_{dyn}^{2}+{Surge}_{dyn}^{2}+{Sway}_{dyn}^{2}}$ Eq.3

Where VeDBA = Vectorial Dynamic Body Acceleration (*g*), Heave_dyn_ = Dynamic acceleration value in heave axis (*g*), Surge_dyn_ = Dynamic acceleration value in surge axis (*g*), Sway_dyn_ = Dynamic acceleration value in sway axis (*g*).

$VeSBA= \sqrt{{Heave}_{stat}^{2}+{Surge}_{stat}^{2}+{Sway}_{stat}^{2}}$ Eq.4

Where VeSBA = Vectorial Static Body Acceleration (*g*), Heave_stat_ = Static acceleration value in heave axis (*g*), Surge_stat_ = Static acceleration value in surge axis (*g*), Sway_stat_ = Static acceleration value in sway axis (*g*).

$Anim.stat= \sqrt{\left( 1-VeSBA \right)^{2}}$ Eq.5

Where Anim.stat = Animal Static Acceleration (*g*), VeSBA = Vectorial Static Body Acceleration (*g*).

$Pitch=atan\left( \frac{{Surge}_{corr}}{\left( {Sway}_{corr}^{2}+{Heave}_{corr}^{2} \right)^{0.5}} \right)\times\left( \frac{180}{\pi} \right)$ Eq.6

$Roll=atan\left( \frac{{Sway}_{corr}}{\left( {Surge}_{corr}^{2}+{Heave}_{corr}^{2} \right)^{0.5}} \right)\times\left( \frac{180}{\pi} \right)$ Eq.7

Where Surge_corr_ = corrected surge acceleration (*g*), Sway_corr_ = corrected sway acceleration (*g*), Heave_corr_ = corrected surge acceleration (*g*).

**References**

[S1] GCDC. <http://www.gcdataconcepts.com/GCDC_X8M-3_User_Manual.pdf>. 2012. Accessed 22/09/2020.

**Supplementary information – Figure legends**

**Fig. S1: Diagrams of the exercise arena and collar design. a)** Diagrammatic representation of collar design. A=Collar with buckle in black; B=Devices (see C for detailed description); (+)=positive acceleration in given axis; (-)=negative acceleration in given axis; Inset C: grey shaded areas=steel nuts; acc=accelerometer devices, stacked atop each other; s.a.t.=self-amalgamating tape, securing nuts and devices to collar; t.t.=Tesa tape, adding additional security and protection to devices. **b)** Aerial view of the arena in which cheetahs were exercised. Black line=course on which lure travelled; brown circles=termite mounds; Grey lines=perimeter fences.

**Fig. S2: Graphic indicating the change in static acceleration (grey arrow). a)** Cheetah standing upright with static acceleration affecting the heave (vertical) axis e.g. giving a value of 1*g*. **b)** Cheetah with head pointed towards the ground, resulting in static acceleration registering in the surge (longitudinal) axis e.g. giving a value of 1*g*. **c)** Cheetah lying on back with static acceleration affecting the heave (vertical) axis e.g. giving a value of -1*g* (opposite of scenario a).
