# Supplementary figures and images for: Categorising cheetah behaviour using tri-axial accelerometer data loggers: a comparison of model resolution and data logger performance
Source: Mov Ecol. 2022 Feb 5;10:7. doi: 10.1186/s40462-022-00305-w (PMC8818224; doi:10.1186/s40462-022-00305-w)

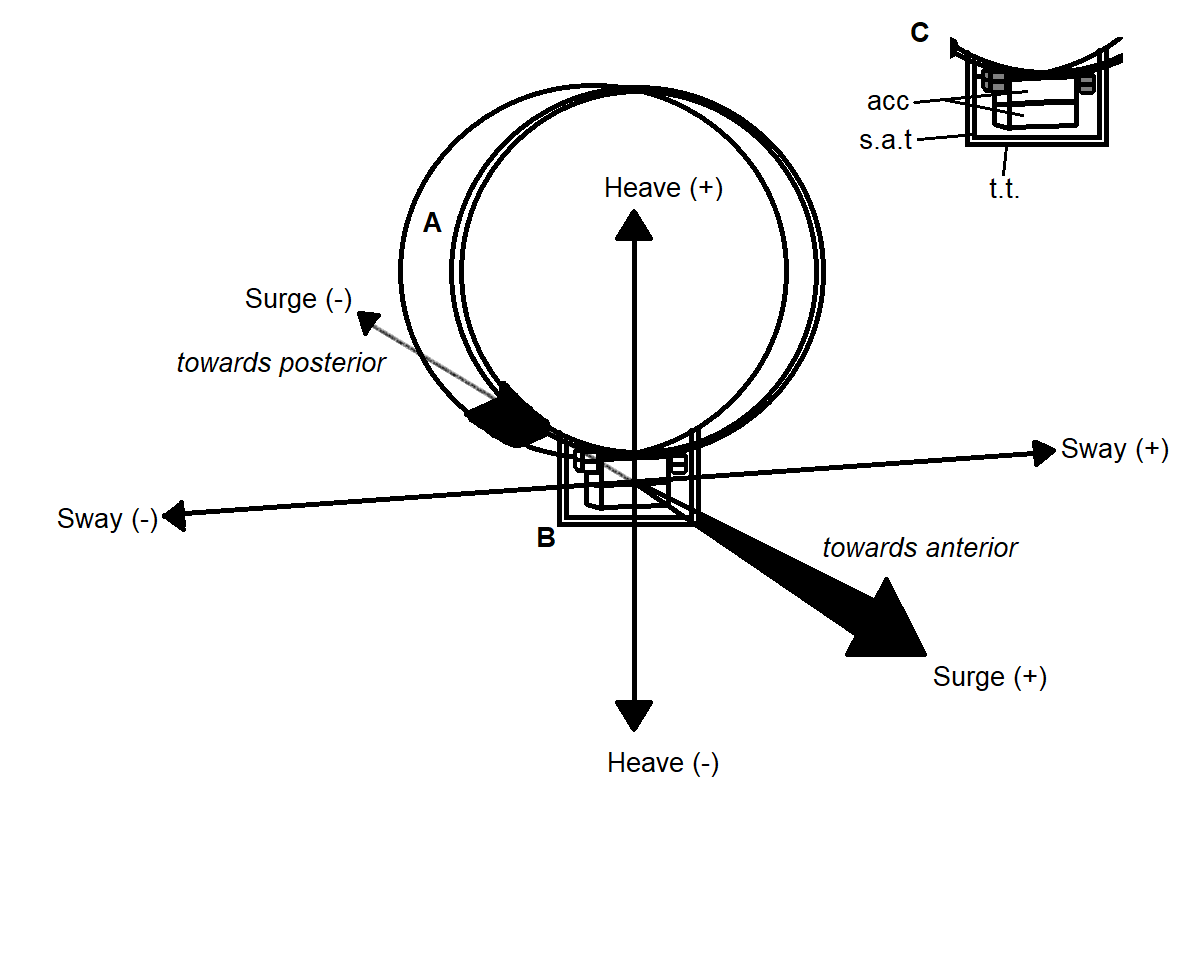

Supplement: Supplementary file 3 — Additional file 3: Diagrammatic representation of collar design. A = collar with buckle in black; B = devices (see C for detailed descripton); (+) = positive acceleration in given axis; (-) = negative acceleration in given axis; Inset C: grey shaded areas = steel nuts; acc = accelerometer devices stacked atop each other; s.a.t. = self-amalgamating tape, securing nuts and devices to collar; t.t. = Tesa tape, adding additional security and protection to devices. [file 40462_2022_305_MOESM3_ESM.tif]

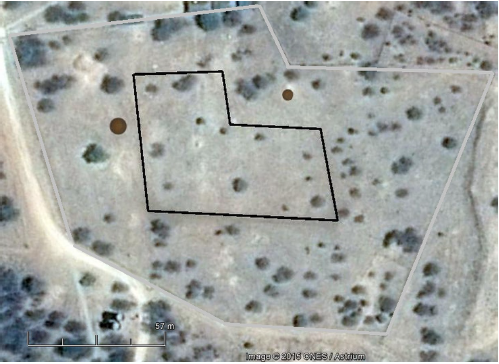

Supplement: Supplementary file 4 — Additional file 4: Aerial view of the arena in which cheetahs were exercised. Black line = course on which lure travelled; brown circles = termite mounds; grey lines = perimeter fences. [file 40462_2022_305_MOESM4_ESM.tif]

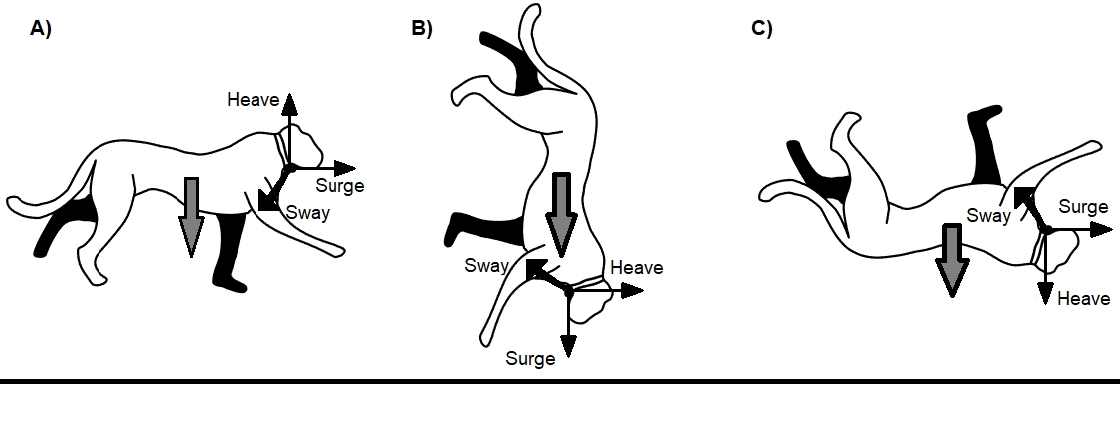

Supplement: Supplementary file 5 — Additional file 5: Graphic indicating the change in static acceleration (grey arrow). a) Cheetah standing upright with static acceleration affecting the heave (vertical) axis e.g. giving a value of 1 g. b) Cheetah with head pointed towards the ground, resulting in static acceleration registering in the surge (longitudinal) axis e.g. giving a value of 1 g. c) Cheetah lying on back with static acceleration affecting the heave (vertical) axis e.g. giving a value of -1 g (opposite of scenario a). [file 40462_2022_305_MOESM5_ESM.tif]
